# A binge high sucrose diet provokes systemic and cerebral inflammation in rats without inducing obesity

**DOI:** 10.1038/s41598-021-90817-z

**Published:** 2021-05-27

**Authors:** Omkar L. Patkar, Abdalla Z. Mohamed, Ashwin Narayanan, Karine Mardon, Gary Cowin, Rajiv Bhalla, Damion H. R. Stimson, Michael Kassiou, Kate Beecher, Arnauld Belmer, Ignatius Alvarez Cooper, Michael Morgan, David A. Hume, Katharine M. Irvine, Selena E. Bartlett, Fatima Nasrallah, Paul Cumming

**Affiliations:** 1 Macrophage Biology Group, Mater Research, Translational Research Institute, Brisbane, QLD Australia; 2 Queensland Brain Institute, The University of Queensland, Brisbane, QLD Australia; 3 Queensland University of Technology, Translational Research Institute, Brisbane, QLD Australia; 4 Centre for Advanced Imaging, The University of Queensland, Brisbane, QLD Australia; 5 Department of Anatomy and Neuroscience, The University of Melbourne, Melbourne, Australia; 6 Department of Nuclear Medicine, Inselspital, Bern University, Bern, Switzerland; 7 School of Psychology and Counselling, Queensland University of Technology, Brisbane, Australia; 8 School of Chemistry, University of Sydney, Sydney, NSW 2006 Australia

**Keywords:** Glial biology, Neuroimmunology, Neuroscience

## Abstract

While the dire cardiometabolic consequences of the hypercaloric modern ‘Western’ diet are well known, there is not much information on the health impact of a high sucrose diet not inducing weight gain. Here, we tested the hypothesis that rats reared with intermittent binge access to sucrose in addition to normal chow would develop an inflammatory response in brain. To test this hypothesis, we undertook serial PET/MRI scans with the TSPO ligand [^18^F]DPA714 in a group of (n=9) rats at baseline and again after voluntarily consuming 5% sucrose solution three days a week for three months. Compared to a control group fed with normal chow (n=9), the sucrose rats indeed showed widespread increases in the availability of cerebral binding sites for the microglial marker, despite normal weight gain compared to the control diet group. Subsequent immunofluorescence staining of the brains confirmed the PET findings, showing a widespread 20% increase in the abundance of IBA-1-positive microglia with characteristic ‘semi-activated’ morphology in the binge sucrose rats, which had 23% lower density of microglial endpoints and 25% lower mean process length compared to microglia in the control rats with ordinary feeding. GFAP immunofluorescence showed no difference in astroglial coverage in the sucrose rats, except for a slight reduction in hypothalamus. The binge sucrose diet-induced neuroinflammation was associated with a significant elevation of white blood cell counts. Taking these results together, we find that long-term intake of sucrose in a binge paradigm, similar in sucrose content to the contemporary Western diet, triggered a low-grade systemic and central inflammation in non-obese rats. The molecular mechanism of this phenomenon remains to be established.

## Introduction

There is a growing concern about the effects of the hypercaloric western diet on cognitive health and aging. The Prospective Urban Rural Epidemiology (PURE) study links excessive intake of carbohydrates, an important aspect of the contemporary Western diet, to increased mortality^1^. While the pro-inflammatory aspects of metabolic syndrome and incipient type 2 diabetes are well established, there is less evidence for activation of inflammatory pathways by an a high sugar diet not inducing obesity^2^. Sucrose, a disaccharide of glucose and fructose, has come to represent a significant caloric component of the Western diet, and excessive dietary fructose is a lipogenic inducer of non-alcoholic fatty liver disease (NAFLD)^3^. A hypercaloric diet high in sucrose or high fructose corn syrup increased interleukin-1β levels in rat liver, and interfered in the rats' performance of a spatial learning task, suggesting a systemic inflammatory response^4^ resulting in cognitive effects reminiscent of those provoked by bacterial lipopolysaccharide (LPS) treatment^5,6^.

There is an abundance of evidence that peripheral factors can influence markers of neuroinflammation in the central nervous system. For example, peripheral treatment with LPS acutely increased the cerebral binding of positron emission tomography (PET) ligands for the 18 kDa translocator protein of (TSPO) in mice^7^ and healthy human volunteers^8^. These TSPO PET findings presumably indicate activation or increased expression of microglia, the resident macrophages in brain^9^. Increased macrophage infiltration in white fat is a major source of inflammatory cytokines in obesity^10^, and inflammatory macrophages imparted high TSPO binding within the aortal lumen of mice with atherosclerosis provoked by high fat diet^11^. However, obese mice did not show any increase in cerebral TSPO binding to [^11^C]PB28 PET^12^, and a binding study in vitro showed a 20% decrease in [^3^H]PK11195 binding in brain membranes from obese rodents^13^, which concurred with the inverse relationship between [^11^C]PBR28 binding with BMI in a large series of humans^14^. Thus, inflammatory responses to obesity may differ in brain and peripheral organs. However, neither clinical nor preclinical PET studies have investigated possible effects of a non-obesity inducing high sucrose diet on cerebral TSPO binding. To isolate effects of dietary sucrose from those potentially arising due to obesity, metabolic syndrome, or type 2 diabetes, we wished to administer sucrose for an extended period of time without altering body weight. In the rodent binge access model, the duration of exposure to a palatable sucrose-rich meal influences the extent of weight gain^15^, and in our hands, intermittent binge access to sucrose in drinking water is titratable to avoid weight gain relative to rats receiving only normal diet and plain water. Therefore, we used the binge sucrose model to test our hypothesis that, in the absence of weight gain, long-term sucrose consumption will provoke inflammatory reactions in blood and brain of rats.

## Materials and methods

### Ethics

All experimental procedures were carried out in accordance with the Australian Code for the Care and Use of Animals for Scientific Purposes. The protocols were approved by the Queensland University of Technology Animal Ethics Committee and the University of Queensland Animal Ethics Committee. The study was carried out in compliance with the ARRIVE guidelines for animals.

### Animals, housing and sucrose intake

Male Wistar rats (ARC, WA, Australia) aged five weeks at arrival were individually housed in open-topped cages, with provision of standard rat chow (Purina; fat 5%, protein 19%, carbohydrates 69% cholesterol 0, fructose 0, glucose 0) and water available ad libitum. We adapted the Intermittent-Access Two-Bottle Choice Drinking Paradigm (TBC)^16^ to allow the rats access to sucrose. On the first day of the 12-week sucrose experimental period, we randomly assigned the rats to one of two groups. The rats in the sucrose diet group (n = 9) had access to one bottle of 5% (w/v) sucrose in water and another bottle with water on Mondays, Wednesdays, and Fridays for 24 h each day. The rats in the normal diet group (n = 9) had access to two bottles of water on the same days. The left–right placement of the sucrose and the water bottles was alternated on each exposure to control for side preferences. The fluids were presented in 300 ml graduated plastic bottles with stainless-steel drinking spouts. All bottles were weighed to the nearest 0.1 gram before and after presentation for 24 h. The rats were weighed three times a week to calculate the grams of sucrose intake per kilogram of body weight. All rats had unlimited access to water and normal diet on all other days.

### Synthesis of [^18^F]DPA714

[^18^F]DPA714 was synthesized by nucleophilic fluorination of toluene-4-sulfonic acid 2-[4-(3-diethylcarbamoylmethyl-5,7-dimethyl-pyrazolo-[1,5-a]pyrimidin-2-yl)-phenoxy]-ethyl ester with [^18^F]fluoride (15 to 20 GBq) on a Synthra RN-Plus unit using a modified procedure previously described by James et al.^17^. [^18^F]DPA714 was purified by HPLC and reformulated using a C18 column to give a final formulation in 10% EtOH and 90% saline.

### PET/MRI scanning

**Figure 1:**
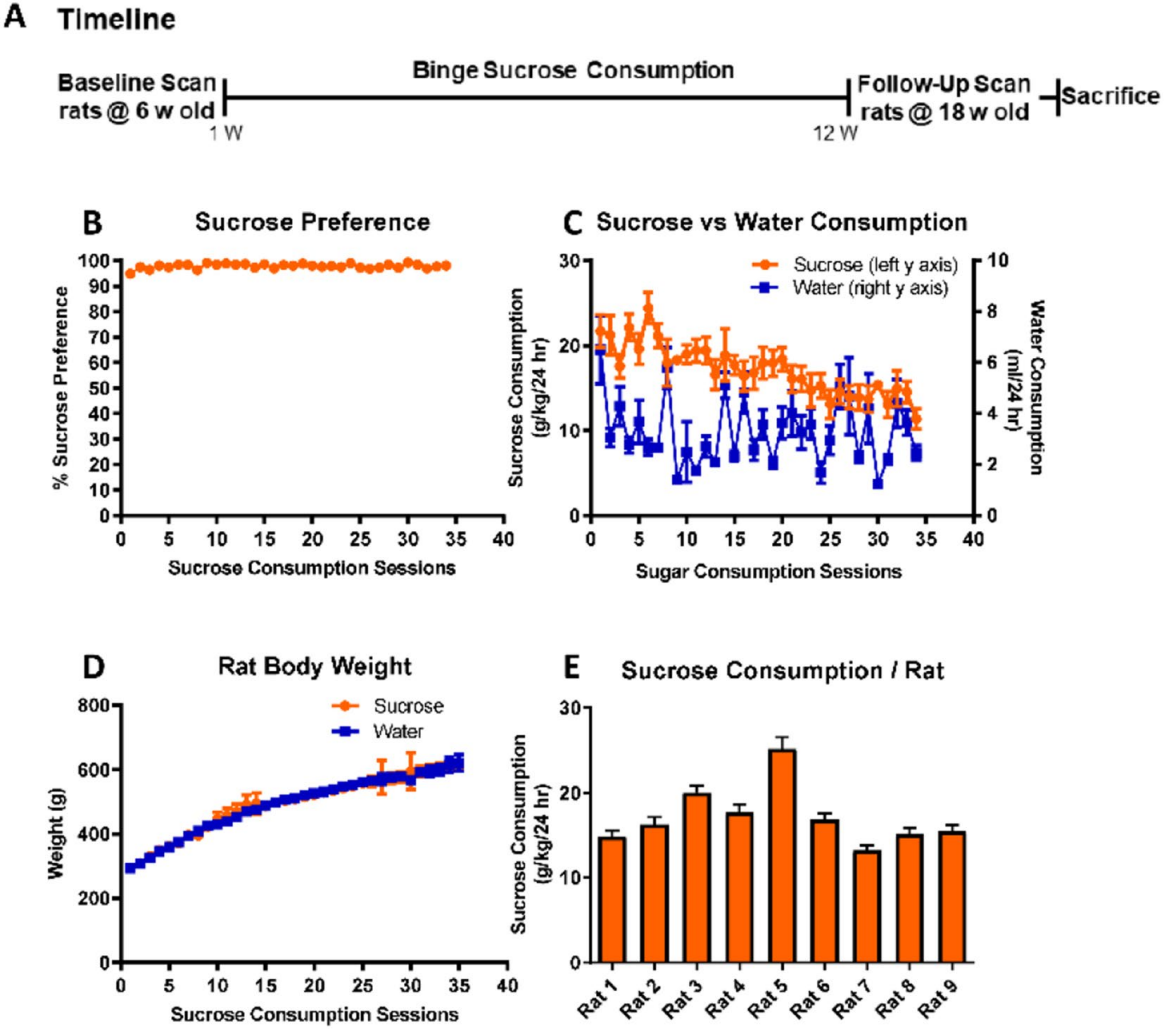
Study timeline and 12-week binge sucrose intake in rats. (**A**) Experimental timeline showing MRI/PET scanning before and after 12-week sucrose/normal diet in rats. All rats resumed the TBC for one week, whereupon they were deeply anesthetized and bled for haematology analysis followed by brain harvesting upon transcardial perfusion with 4% PFA. (**B**) Mean sucrose preference (%) ± SEM shows a high preference in the rats for sucrose water over plain water when presented with a choice to consume either. (**C**) Mean sucrose (g/kg/24 h) ± SEM and mean water (ml/24 h) ± SEM intake for rats in the TBC over 12 weeks or 36 drinking sessions. (**D**) Mean body weight (g) ± SEM of the rats in the sucrose and normal diet group shows no significant differences between the two over 12 weeks. (**E**) Individual mean sucrose (g/kg/24 h) intake ± SEM of all rats in the sucrose diet group. All values presented are from n = 9.

All rats in the sucrose diet (n = 9) and normal diet (n = 9) groups were scanned using [^18^F]DPA714 at (baseline; 6 weeks old) just prior to the binge sucrose protocol, and again (follow-up) after 12 weeks of the sucrose intake protocol, when they were 18 weeks old (Fig. 1a). On each scanning day, rats were anesthetized with isoflurane (2%) delivered in oxygen by a facemask. A thin catheter was placed in a tail vein for administration of radiotracer, and the rats were positioned within the aperture of the combined MRI/PET system. This was a 300 mm bore 7 T ClinScan, running Siemens VB17, equipped with a removable PET insert containing three rings of 16 detector blocks with 15 × 15 LSO crystals (1.6 × 1.6 × 10 mm) per block, placed at the centre of the magnet bore, and operating by the Siemens Inveon Acquisition Workplace (IAW) (Bruker, Germany). A 40 mm ID rat head MRI radio frequency coil inside the PET ring was used to acquire head images simultaneously with the dynamic PET acquisition. Rats were injected with approximately 30 MBq of [^18^F]DPA714 formulated in saline to a total volume of 400 µl, and administered as a slow intravenous bolus beginning at the start of the dynamic PET-MR recording. The dynamic PET emission recording consisted of ten frames of one min each followed by ten frames of five min duration, thus to a total of 60 min. During the first phase of the PET recording, animals were MR scanned using T1 map TR/TE/flip angle (FA) = 12 ms/1.02 ms/4° and 21°, isotropic resolution 0.32 mm.

### PET/MRI analysis

The dynamic PET data was reconstructed using dedicated PET reconstructed software developed at the University of Tubingen. The attenuation and decay corrected PET images were reconstructed with a final matrix of 128 × 128 × 89 using the ordered-subset expectation maximization. The standard uptake value (SUV) maps were calculated based on the injected dose and animal weights. The SUV frames were linearly co-registered to correct for motion artifact. To register the data to a common space, each rat`s individual T1 image was affine registered to an anatomic rat brain atlas reference template using the program REGISTER (Montreal Neurological Institute) with nine degrees of freedom. The calculated transformation matrix was then applied to each of the SUV frames to register the SUV maps to the anatomic rat brain atlas reference template. The SUV maps from all animals were averaged and a cerebellum VOI mask was generated by applying the standard cerebellum anatomic template to the average SUV template, which was then degraded by setting a 75% intensity threshold to avoid spill-in due of progressive accumulation of cranial signal. The new study-specific cerebellum reference mask was applied to the dynamic sequence, after confirming good placement in the resampled PET summation image. Voxel-wise parametric maps of binding potential (BP_ND_) were calculated by the Logan method, using the cerebellum as a reference tissue^18^, with data recorded from 10 to 60 min used for the linearization.

### Blood profiling

After completing the 12-week follow-up scans, rats returned to their home cages and resumed their previous treatment for one week, whereupon they were deeply anesthetized with 4–5% isoflurane prior to collection of tail blood (~ 20 µl) into heparinized capillary tubes. A complete blood count was undertaken using a Mindray BC-5000 Vet Auto Haematology Analyser (Shenzhen, China).

### Brain harvesting and sectioning

Following the final blood collection, the rats were transcardially perfused with 4% paraformaldehyde in 0.1 M phosphate-buffered saline solution (PBS, pH 7.4) while deeply anesthetized with isoflurane. After their removal, brains were post-fixed overnight at 4 °C and transferred to PBS. Coronal plane sections 40 μm-thick were cut using a vibratome (Leica V1200S) and kept as free-floating sections in PBS azide at 4 °C.

### Brain immunohistochemistry

Immunohistochemistry was performed on rat coronal brain sections collected between bregma − 2.16 to 2.5 according to the atlas of Paxinos and Watson^19^. Immunohistochemical staining was performed as follows: sections were first incubated at room temperature for 30 min in permeabilization buffer (1% Triton-X and 0.1% Tween 20 in PBS) followed by 30 min in blocking solution (4% fetal calf serum, 0.3% Triton-X and 0.05% Tween 20 in PBS). Sections were then incubated for 24 h at room temperature under orbital agitation in either goat anti IBA-1 (Thermofisher Scientific 21247, 1:500) for microglial analysis or rabbit anti GFAP (Dako Agilent Z0334, 1:500) for astroglial analysis, diluted in blocking solution. After 3 × 10 min washes in blocking solution, slices were incubated for 4 h at room temperature either in mouse-anti-goat-Alexa 488 (1/1000) or goat anti-rabbit-Alexa 594 (1:1000), diluted in blocking solution. Slices were then washed in PBS (3 × 10 min) followed by a five min incubation with DAPI (1:5000) (Thermofisher Scientific D1306) diluted in PBS. All sections were washed once with PBS for 5 min and mounted with Fluorescence Mounting Medium (Dako, Agilent).

### Confocal imaging

Confocal images (1024 × 1024) were acquired on an Olympus FV3000 microscope with a 40 × air objective (NA 1.75) equipped with solid state lasers (405 and 488 nm) at an exposure of 2 µs/pixel, a numerical zoom of 1 × , and a z-step of 0.5 µm. We acquired nine images at a z-depth of 10 µm (0.5-µm step size, 20 z-stacks) for each selected brain region from all rats. Immunohistochemistry images were converted into 8-bit files and the z-stack projections were analyzed using FIJI software.

### Image analysis

IBA-1 positive cells were manually counted using FIJI software (National Institute of Health) by an experimenter blinded to the treatment group. These numbers were then normalized to the brain region volume imaged calculated as μm^3^ (imaged area [µm^2^] × no of stacks). For GFAP, we analyzed fluorescence intensity and percentage area of positive staining using the ‘measure’ tool in FIJI software following adjustment of brain region specific threshold, which we kept consistent for all rats.

For microglial ramification analysis, all photomicrographs from different brain regions were processed with appropriate plugins (FFT band-pass filter, unsharp mask, despeckle, remove outliers, and close) in the FIJI software prior to converting them into binary and skeletonized images^20^ (Fig. 5A–D). The skeletonized images were then analyzed in the same software using Analyze Skeleton Plugin (http://imagej.net/AnalyzeSkeleton31), which tags skeletal features relevant to microglia ramification: slab voxels (orange, process length) and endpoints (blue, Fig. 5E). The summarized endpoints and branch lengths were then normalized by the number of microglia cell somas in each image (previously calculated with IBA-1 positive cell counts) to calculate the number of microglia endpoints/cell and microglia process length/cell.

### Liver histology, immunohistochemistry and imaging

In addition to brain, liver samples were harvested from the rats and post fixed in 4% PFA, followed by staining of 4 µm-thick sections with eosin (30 s) and hematoxylin (60 s) (Sigma-Aldrich), and whole-slide digital imaging performed on the VS120 Olympus slide scanner (Olympus-Lifescience, Australia). For liver IBA-1 immunohistochemistry, 40 µm-thick sections were processed and imaged as described above for brain sections.

### Statistics

All data were plotted in Graphpad Prism 6.0 software (La Jolla, California, USA). All numerical data are expressed as mean ± SEM, with significance established at p < 0.05. Group means of PET, immunohistochemistry, and blood cell counting results were compared using one or two-tailed Student’s t-test.

We calculated mean parametric binding potential (BP_ND_) maps for the normal diet and binge sucrose diet groups at baseline and at 12-week follow-up (Fig. 1). To compare between groups and different time points, the data were analyzed by voxel-wise cross-subject statistics by repeated measures analysis of variance (ANOVA) using FSL (FMRIB Library, Oxford, UK), and a paired t-test was performed using nonparametric permutation test (FSL-randomise [2]) with 1000 permutations. The results were corrected for multiple comparisons using family-wise error correction (FWE; α < 0.05). The regions with significant changes in BP_ND_ in the contrast between follow-up scans and baseline scans were used to generate a volume of interest to calculate the mean (SEM) of the BP_ND_ for the normal diet and sucrose diet groups at baseline and at 12 weeks follow-up.

## Results

### Voluntary binge intake of 5% sucrose in addition to normal chow does not cause additional weight gain in rats as compared to normal diet

The rats in the sucrose group, when allowed a choice of 5% sucrose solution or plain water, showed strong preferences for sucrose (97.9 ± 0.15%) (Fig. 1B). The mean weekly sucrose consumption of rats in the sucrose group was 17.2 ± 0.50 g/kg body weight (week 1: 21.7 ± 1.90 g/kg, week six; 18.0 ± 1.80 g/kg, and week 12; 14.6 ± 1.20 g/kg) (Fig. 1C). The mean individual sucrose consumption per rat over the 12-week period is shown in Fig. 1E. Despite their preference for sucrose, mean body weight gains did not differ between the normal diet group (272 ± 4.6 g at baseline to 507 ± 16.7 g at 12 weeks) and sucrose diet group (270 ± 3.7 g at baseline to 501 ± 17.3 g at 12 weeks, *p* = 0.94) (Fig. 1D). We did not weigh the chow consumption, so we cannot confirm whether binge sucrose rats regulated their mean daily caloric intake by reducing their chow consumption, nor do we have information about their physical activity or fat/muscle mass proportions.

### Long-term sucrose diet not inducing obesity triggers central and peripheral inflammation in rats

**Figure 2:**
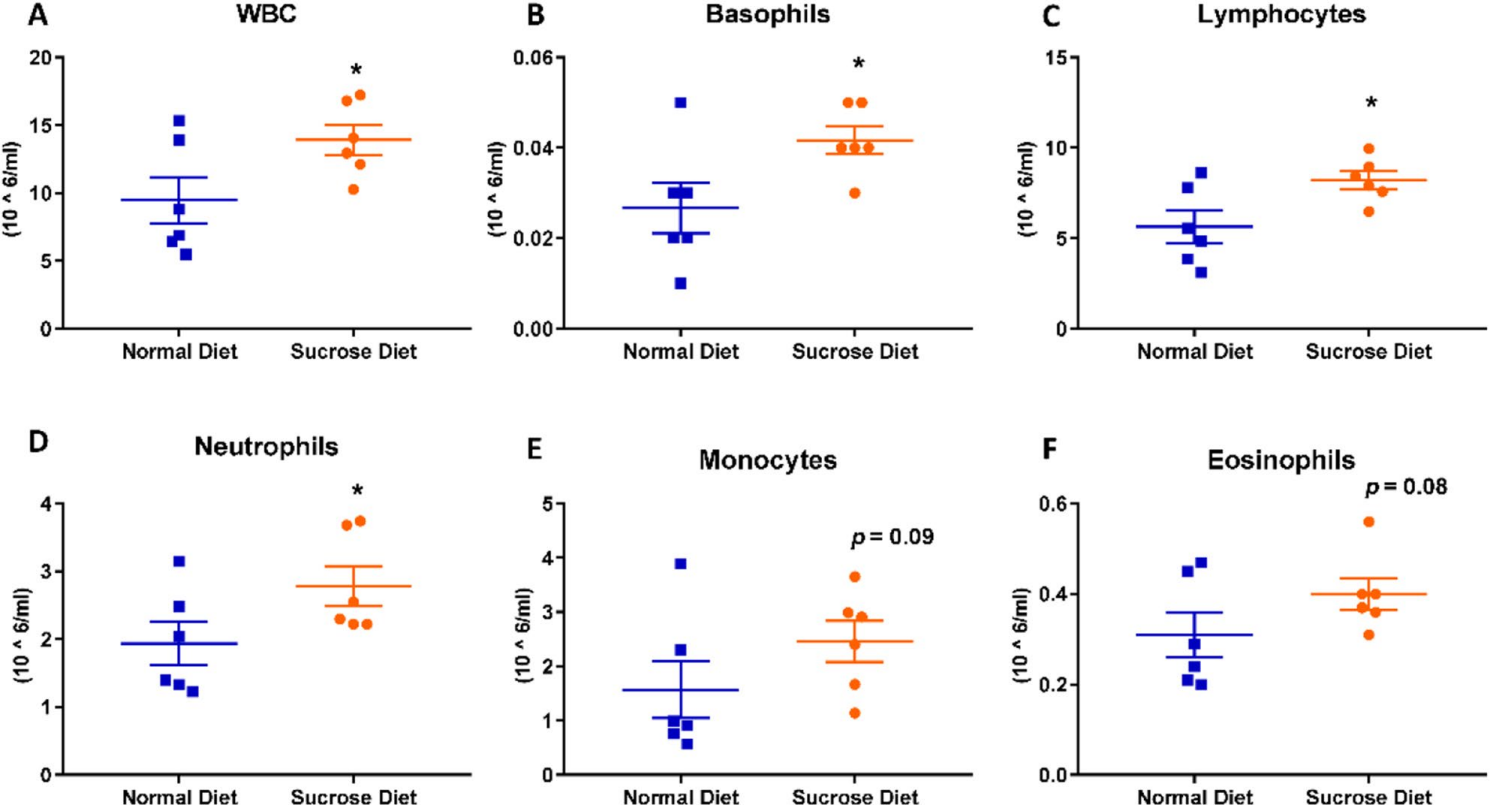
Blood profiling to detect peripheral inflammation following 12-week binge sucrose intake in rats. Blood profiling revealed elevated (**A**) white blood cells, (**B**) basophils, (**C**) lymphocytes and (**D**) neutrophils in rats in the sucrose-diet group as compared to the normal diet group. Increases in (**E**) blood monocytes and (**F**) eosinophils in the sucrose diet rats were not statistically significant. All values are presented as mean cell counts ± SEM (10^6^/ml) analysed with Student’s one-tailed t-test. *p < 0.05 compared with normal diet-fed controls. All values presented are from n = 6.

To analyse the effects of long-term binge sucrose intake on blood leukocyte populations, we drew blood immediately before transcardial perfusion. Blood profiling revealed significantly higher counts of white blood cells (WBC; 9.47 ± 1.69 vs 13.91 ± 1.10, Cohen’s d = 1.29, *p = 0.02, Fig. 2A), basophils (0.02 ± 0.00 vs 0.04 ± 0.00, Cohen’s d = 2.31, *p = 0.02, Fig. 2B), lymphocytes (5.6 ± 0.80 vs 8.2 ± 0.40 Cohen’s d = 1.54, *p = 0.01, Fig. 2C), and neutrophils (1.93 ± 0.30 vs 2.78 ± 0.20, Cohen’s d = 1.10, *p = 0.03, Fig. 2D). There were non-significant increases in monocyte (1.57 ± 0.50 vs 2.46 ± 0.30, p = 0.09, Fig. 2E) and eosinophil counts (0.30 ± 0.00 vs 0.40 ± 0.00, p = 0.08, Fig. 2F) in rats in the binge sucrose diet group.

**Figure 3:**
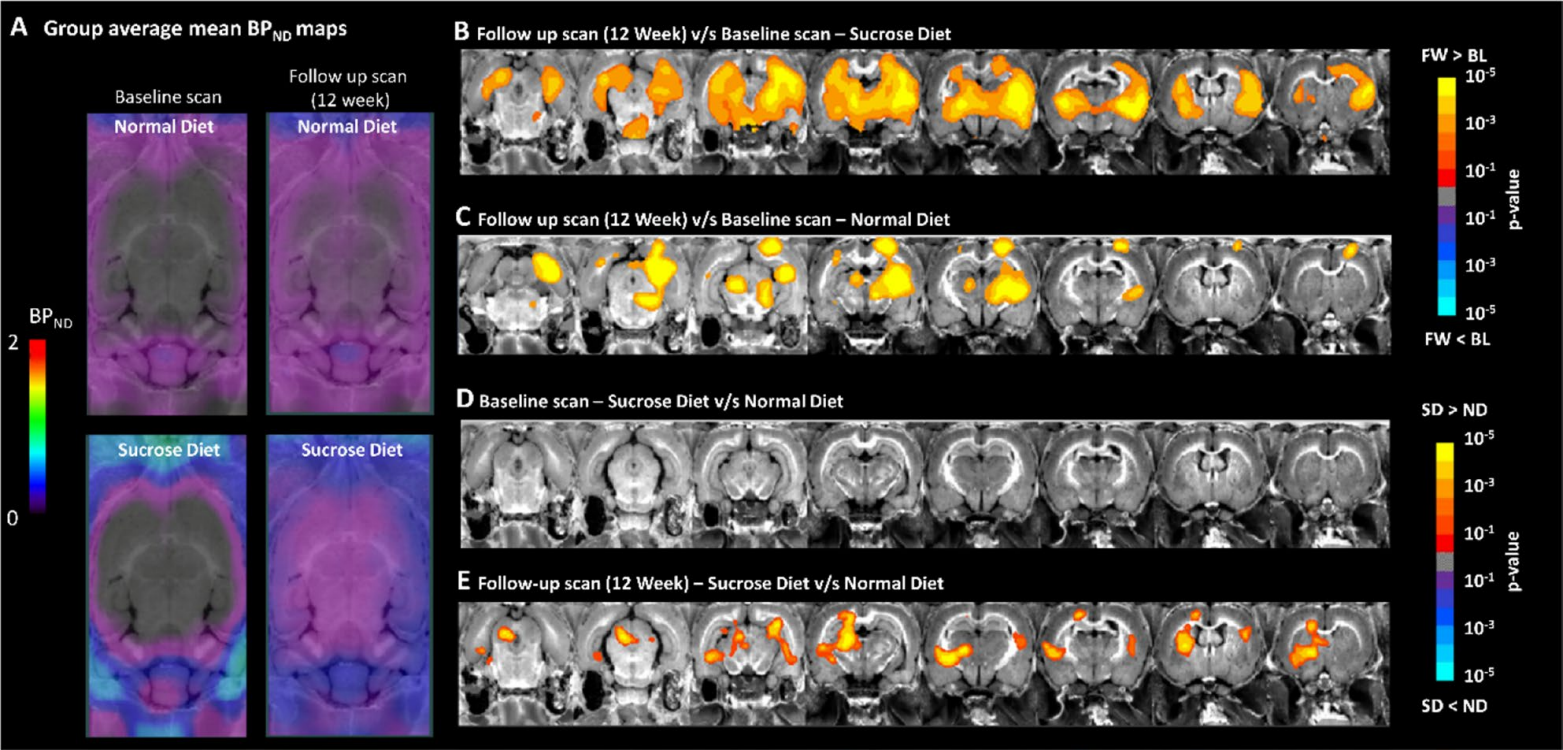
Voxel-wise analysis of effects of binge sucrose diet on the binding potential (BP_ND_) of the TSPO PET tracer [^18^F]DPA714. (**A**) Mean parametric maps of BP_ND_ at baseline and follow-up scans for the rats in the sucrose or normal diet or sucrose diet groups. Each map is the mean of determinations in groups of nine rats. The voxel-based analysis showed increased BP_ND_ of the PET tracer [^18^F]DPA714 in the follow up scan as compared to baseline in (**B**) sucrose diet and (**C**) normal diet groups, showing greater increases in the sucrose diet group. When comparing between sucrose diet and normal diet groups, the data showed (**D**) no differences at the baseline scans between the animals, while (**E**) increased TSPO expression as reflected by the greater [^18^F]DPA714 BP_ND_ in the sucrose diet group compared to the normal diet group at the follow up scans. The results shown here are corrected for multiple comparisons using the family-wise error correction method (FEW; p < 0.05). *FL* follow up, *BL* baseline, *SD* sucrose diet, *ND* normal diet.

We obtained PET recordings with the TSPO tracer [^18^F]FDPA714 in all rats at baseline and 12 weeks follow-up. Mean parametric maps indicate very low baseline BP_ND_ in both groups and give the impression of a globally increased BP_ND_ in the follow-up scans of the binge sucrose group (Fig. 3A). The contrast between baseline and follow-up scans in the binge sucrose group showed widespread BP_ND_ increases (Fig. 3B): motor cortex (0.08 ± 0.00 vs 0.24 ± 0.00, + 200%, Cohen’s d = 3.53, p < 0.001), hippocampus (0.12 ± 0.00 vs 0.31 ± 0.00, + 158%, Cohen’s d = 6.33, p < 0.001), thalamus (0.14 ± 0.00 vs. 0.28 ± 0.07; + 100%, Cohen’s d = 2.60, p < 0.001), and caudate-putamen (0.17 ± 0.00 vs 0.37 ± 0.00, + 118%, Cohen’s d = 2.98, p < 0.001). The same contrast in the normal diet group showed relatively circumscribed clusters of increased [^18^F]DPA714 BP_ND_ (Fig. 3C): motor cortex (0.07 ± 0.00 vs 0.17 ± 0.00, + 142%, Cohen’s d = 2.50, p < 0.001), hippocampus (0.13 ± 0.00 vs 0.25 ± 0.00, + 92%, Cohen’s d = 3.30, p < 0.001) and thalamus (0.13 ± 0.00 vs 0.17 ± 0.00, + 31%, Cohen’s d = 2.50, p = 0.03), and caudate-putamen (0.15 ± 0.00 vs 0.17 ± 0.00, + 13%, p > 0.05). The BP_ND_ maps did not differ between the two groups at baseline (Fig. 3D), but at follow-up there were voxel clusters where BP_ND_ in the sucrose diet group exceeded that in the normal diet group (Fig. 3E): left hippocampus (p < 0.001), right hippocampus (p = 0.01), motor cortex (p = 0.001), thalamus (p < 0.001), and caudate-putamen (p < 0.001).

### Long-term sucrose diet not inducing obesity triggers microgliosis in the rat brain

**Figure 4:**
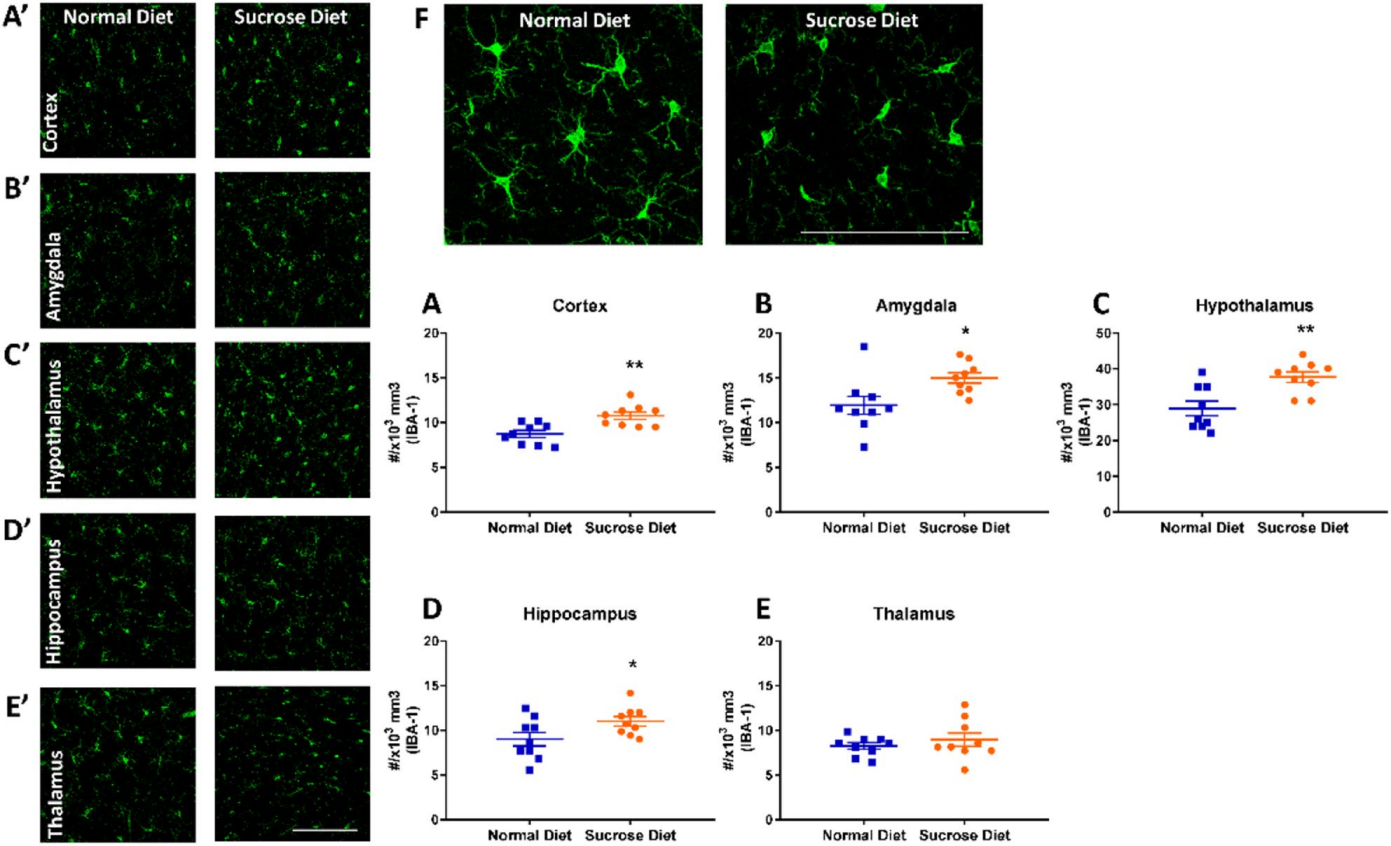
Quantification of microglia following 12-weeks binge sucrose intake in rats. Immunohistological analysis revealed elevated microglial cell count in the (**A**) motor cortex, (**B**) amygdala, (**C**) hypothalamus and (**D**) hippocampus in the sucrose diet rats as stained with IBA-1 (green). (**A’**–**D’**) shows representative staining in each of these regions in the normal diet (left panel) and sucrose diet (right panel) groups, scale bar = 100 µm. The density of microglia did not differ in the thalamus (**E** and **E’**), scale bar = 100 µm. All values are presented as mean number of IBA-1 positive cells ± SEM normalized to volume of the brain region imaged (10^3^ mm^3^) analysed with Student’s two-tailed t-test. *p < 0.05, **p < 0.01 compared to controls. All values presented are from n = 9 per group.

Following resuming sucrose diet or normal diet access after the follow-up PET/MRI scan at 12 weeks, all rats were transcardially perfused with 4% PFA, and their brains harvested and processed for immunohistological detection of neuroinflammation. IBA-1 staining revealed in the binge sucrose rats a global increase in the microglial density (× 10^3^/mm^3^) in the motor cortex (8.7 ± 0.38 vs 10.8 ± 0.40, Cohen’s d = 1.78, **p = 0.002, Fig. 4A), amygdala (11.9 ± 1.00 vs 15.0 ± 0.57, Cohen’s d = 1.25, *p = 0.018, Fig. 4B), hypothalamus (28.9 ± 2.03 vs 37.7 ± 1.47 Cohen’s d = 1.66, **p = 0.003, Fig. 4C) and hippocampus (9.0 ± 0.76 vs 11.0 ± 0.53, Cohen’s d = 1.16 *p = 0.04, Fig. 4D). There was, however, no group difference in IBA-1 density in the thalamus (8.3 ± 0.36 vs 9.0 ± 0.74, p = 0.40, Fig. 4E). The increased microglial density in brains of rats on the sucrose diet was accompanied by changes in microglial morphology, with reduced ramification as compared to the rats fed on normal diet (Fig. 4F).

### Long-term sucrose diet-associated microgliosis is accompanied by morphological alterations in microglial ramification

Microglial morphology is a reliable indicator of microglial activation; normal resting microglia have ramified processes, while activated microglia in altered physiological states, diseases and inflammation have reduced ramifications^21,22^. To quantify the observed decreased in microglial ramification in the sucrose group rats, we measured microglial endpoints and total branch length from entire photomicrograph fields (not individual cells) from different brain regions. We normalized the results to the number of Iba-1 positive microglial cell somata to calculate the number of microglia endpoints/cell and microglia process length/cell.

**Figure 5:**
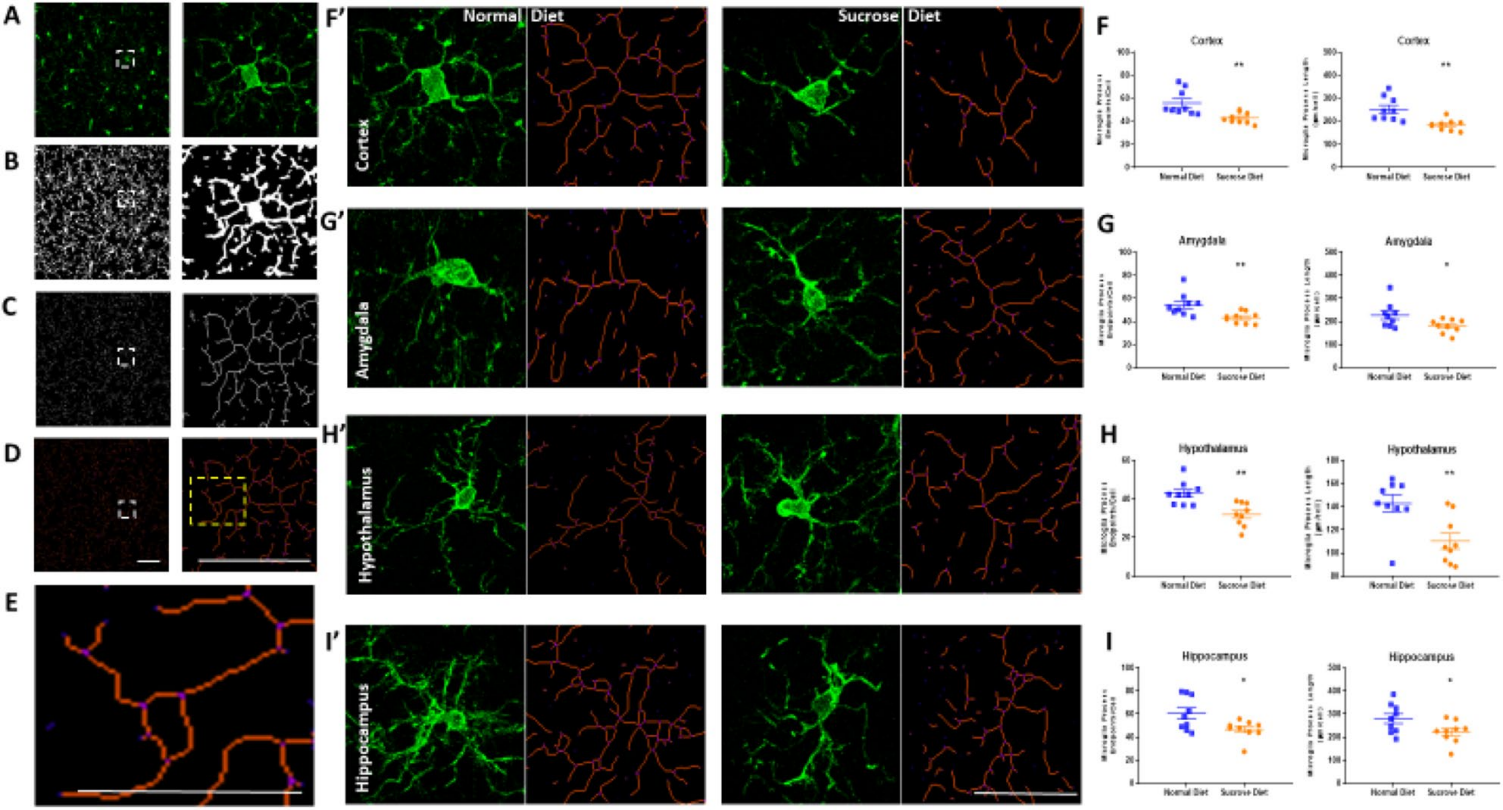
Analysis of microglial morphology following 12-week binge sucrose intake in rats. Microglial morphology was analysed in Fiji software using the ‘Analyse Skeleton’ plugin. (**A**–**D** left panel) shows the process to prepare photomicrographs for skeleton analysis using a series of plugin protocols, scale bar = 50 µm. (**A**–**D** right panel) shows a magnified version of the white dotted box in (**A**–**D**) left panel, scale bar = 50 µm. (**E**) Magnified version of yellow dotted box in D right panel showing tag skeletonized processes as orange, endpoints as blue, and junctions as purple, scale bar = 25 µm. (**F’**–**I’**) Representative skeletonized image and original photomicrograph of a single IBA-1 positive cell showing the relationship between skeleton and photomicrograph in a normal diet and a sucrose diet rat (**F’**) motor cortex, (**G’**) amygdala, (**H’**) hypothalamus and (**I’**) hippocampus. Quantification of microglial ramification was performed on entire photomicrographs (not single cells), which showed reduced endpoints/cell and process length/cell respectively in sucrose rat (**F’**) motor cortex, (**G’**) amygdala, (**H’**) hypothalamus and (**I’**) hippocampus, scale bar = 50 µm. All values are presented as number of microglial endpoints/cell ± SEM or total process length/cell ± SEM in the brain region imaged and analysed with Student’s two-tailed t-test. *p < 0.05, **p < 0.01 compared to controls. All values presented are from n = 9 per group.

Our analysis indicated that microglial ramifications were significantly lower in the sucrose group rats as demonstrated by branch endpoints/cell in the motor cortex (56.0 ± 3.6 vs 42.9 ± 1.5, Cohen’s d = 1.54, **p = 0.004, Fig. 5F), amygdala (54.6 ± 3.2 vs 43.5 ± 1.6, Cohen’s d = 1.43, **p = 0.007, Fig. 5G), hypothalamus (42.9 ± 2.0 vs 32.0 ± 2.0, Cohen’s d = 1.79, **p = 0.001, Fig. 5H) and hippocampus (60.6 ± 4.8 vs 46.8 ± 2.7, Cohen’s d = 1.17, *p = 0.023, Fig. 5I). Also, total microglial process lengths were lower in the sucrose group rats as shown by process length/cell (µm/cell) in the motor cortex (253 ± 17 vs 185 ± 8, Cohen’s d = 1.70, **p = 0.002, Fig. 5F), amygdala (228 ± 18 vs 181 ± 10, Cohen’s d = 1.06, *p = 0.03, Fig. 5G), hypothalamus (143 ± 7 vs 110 ± 7, Cohen’s d = 2.04, **p = 0.004, Fig. 5H) and hippocampus (280 ± 21 vs 223 ± 16, Cohen’s d = 1.17, *p = 0.045, Fig. 5I).

### Long-term sucrose diet has no effect on astrocytes in different rat brain regions except hypothalamus

**Figure 6:**
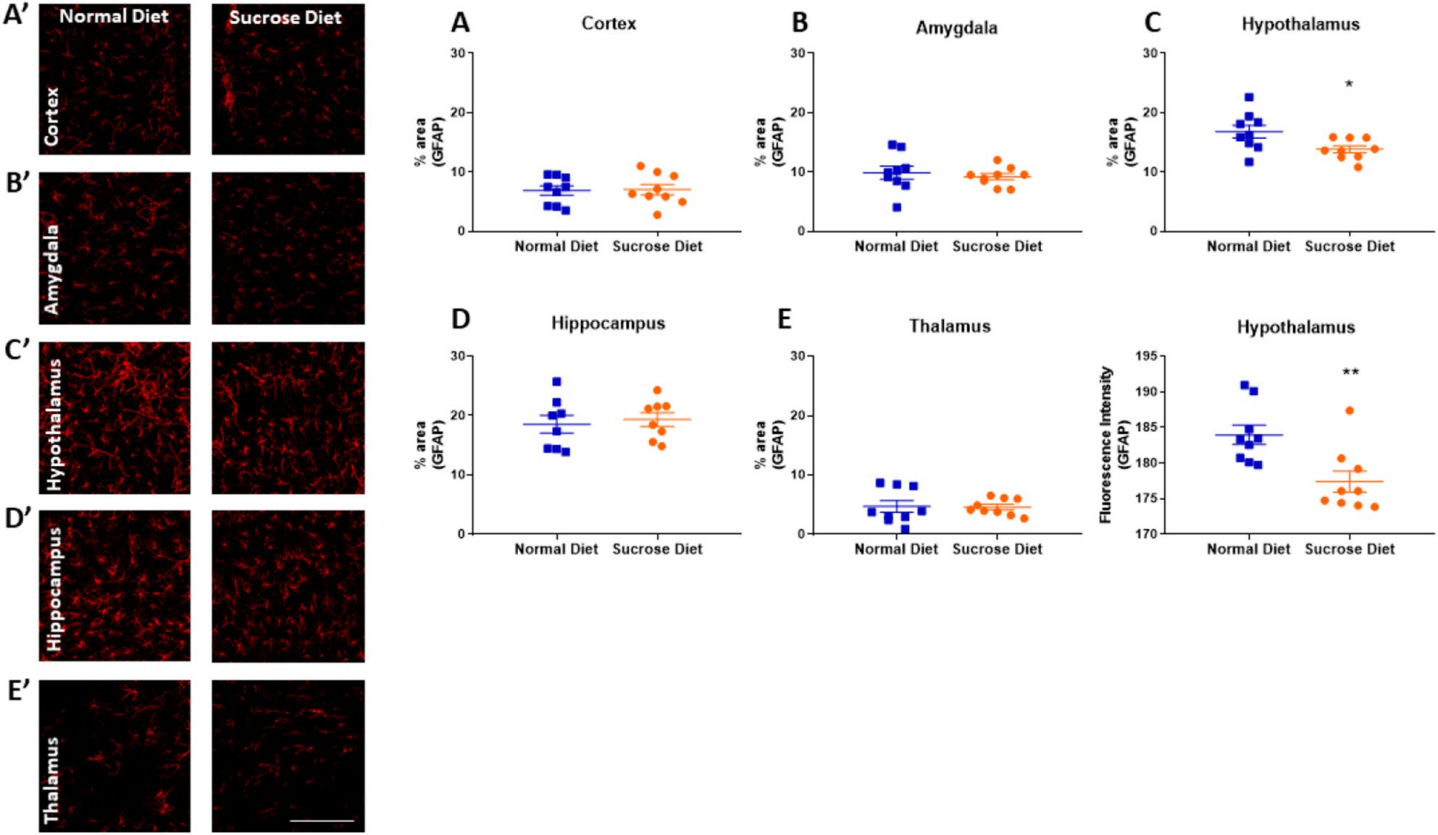
Effect of 12-week binge sucrose intake on astroglial cells in rats. Immunohistological analysis revealed no effect of sucrose diet on the astrocyte coverage in the (**A**) motor cortex, (**B**) amygdala, (**D**) hippocampus and (**E**) thalamus as stained with GFAP (red). (**A’**–**B’** and **D’**–**E’**) shows representative staining in each of these regions in the normal diet (left panel) and sucrose diet (right panel) groups, scale bar = 100 µm. (**C**) The area covered and (**F**) fluorescence intensity of astrocytes staining was reduced in the hypothalamus of the sucrose diet rats. (**C’**) Representative staining of the astroglial marker GFAP in the hypothalamus in the normal diet (left panel) and sucrose diet (right panel) groups, scale bar = 100 µm. All values are presented as mean area covered with GFAP positive staining ± SEM or mean GFAP fluorescence intensity ± SEM in the brain region imaged analysed with Student’s two-tailed t-test. *p < 0.05, **p < 0.01 compared to controls. All values presented are from n = 9 per group.

The sucrose diet had no effect on percentage astrocytic coverage in the motor cortex (6.9 ± 0.79 vs 7.0 ± 0.87%, p = 0.88, F.ig 6A), amygdala (9.9 ± 1.07 vs 9.2 ± 0.52%, p = 0.50, Fig. 6B), hippocampus (19.0 ± 1.51 vs 19.4 ± 1.17%, p = 0.68, Fig. 6D) and thalamus (4.7 ± 0.97 vs 4.6 ± 0.45%, p = 0.92, Fig. 6E) compared to normal diet-fed rats. However, the rats on the sucrose diet had a lower percentage area covered by GFAP in hypothalamus (16.8 ± 1.07 vs 13.8 ± 0.58%, Cohen’s d = 1.15, *p = 0.02, Fig. 6C) and lower GFAP fluorescence intensity compared to the normal diet-fed controls (184 ± 1.4 vs 177 ± 1.5 %, Cohen’s d = 1.56 **p = 0.004, Fig. 6F).

### Assessment of liver for inflammation in the sucrose-diet rats

To investigate the source of peripheral inflammation, we examined the livers of rats fed on normal and binge sucrose diets. H&E staining of sucrose diet-fed rat livers showed no gross difference in histology, no inflammatory infiltration, nor any discernible abnormal fat deposition as compared to normal diet-fed controls (Fig. S1, top two panels). Furthermore, IBA-1 staining of the livers also showed no elevated macrophage density or infiltration in the sucrose diet group (Fig. S1, last panel).

## Discussion

Sucrose or high-fructose corn syrup form the caloric basis of contemporary high selling sugary foods and beverages, together contributing to approximately 50% of the added sugars in the Western diet^23^. Increased public awareness of the cardiovascular and metabolic outcomes of excessive sugar consumption have led to declining sugar intake, although recent statistics show that inhabitants of developed countries still consume an average of at least one sugary beverage per day^24,25^, and that sugar use is burgeoning in developing countries^26^. In this study, we investigated the consequences of three months exposure of rats to high sucrose diet not causing any excessive increase in body weight on brain and body health. As intended, the rats that voluntarily consumed 5% sucrose solution three days a week for three months (along with ad libitum access to normal chow and water) did not gain additional weight compared to their control-fed counterparts. Rats fed with ordinary chow typically consume 180 kcal/Kg body weight per day^27^, whereas rats in this study were consuming 15 g sucrose on binge days, corresponding to 120 kcal/Kg body weight per day; while we did not measure chow consumption, we suppose that the majority of their caloric intake was as sucrose on binge days. While not gaining excess weight, these sucrose-fed rats showed overt signs of neuroinflammation as evidenced by longitudinal TSPO PET imaging and confirmatory immunohistochemical detection of microgliosis.

Low level constitutive expression of TSPO is seen in normal brains, but increased expression of TSPO is a well-established index of neuroinflammation used in many clinical and preclinical molecular imaging studies^28–30^. Among the many available TSPO PET tracers^9^, [^18^F]DPA714 has been used in clinical studies of Alzheimer’s disease^28^, temporal lobe epilepsy^31^, multiple sclerosis^32^, and other disorders. Increased TSPO binding is generally thought to be indicative of reactive microgliosis, although the [^18^F]DPA714 PET signal may be of mixed cellular origin, as TSPO is also expressed in endothelium^33^, perivascular macrophages^34^ and astrocytes^35^, and even neurons. However, the present immunohistochemical findings of increased microglia and unchanged or reduced astrocyte numbers support our interpretation that the increased TSPO signal of microglia origin and is indicative of their activation. Indeed, the morphology of IBA-1-stained microglia with reduced number of endpoints and mean process length was consistent with an activated state in the binge sucrose rats, indicative of a low-grade neuroinflammation.

Our serial [^18^F]DPA714 PET examination showed that 12 weeks of binge sucrose diet increased TSPO binding throughout the forebrain, in excess of the apparent increase occurring with age in the control rats. Previous studies in humans and rodent models have likewise reported age-dependent increases in TSPO-PET signal^14,36,37^, although not previously on a time-scale of only three months. The mean [^18^F]DPA714 BP_ND_ maps show modest effects of age- and sucrose diet-dependent changes, which are abundantly clear in the voxel-wise statistical comparisons. Here, the magnitude and spatial extent of voxel-clusters with increasing BP_ND_ are distinctly greater in the rats from the sucrose diet as compared to the normal diet group. These present results stand in contrast to earlier findings that obese mice did not show any increase in cerebral TSPO binding to [^11^C]PB28 PET^12^, or a 20% decrease in [^3^H]PK11195 binding in brain membranes in vitro^13^. Furthermore, there was a weak inverse correlation between the global cerebral distribution volume of [^11^C]PBR28 with body mass index in humans^14^. Thus, the present findings of microglial proliferation and elevated TSPO binding in rats fed a high long-term sucrose diet (without excess weight gain) suggest a neuroinflammatory effect due to sucrose diet as distinct from obesity per se.

How do the present findings of dietary sucrose-dependent microglial activation compare with increases typically reported in frank neuroinflammatory conditions? For example, [^18^F]DPA714 PET sensitively detected increased TSPO binding in brain of rats treated with quinolinic acid, which provokes a massive inflammatory reaction^17^. While baseline BP_ND_ was near zero in another rat [^18^F]DPA714 PET study, BP_ND_ in the substantial nigra increased to 0.3 after viral over-expression of human mutant α-synuclein^38^. The present sucrose diet-induced increases in [^18^F]DPA714 BP_ND_ in rat brain are thus at the low end of the range reported for frank inflammatory conditions. The PET findings were entirely corroborated by the immunohistochemical results, which unequivocally showed widespread 10–20% increases in the microglial density in the hippocampus, amygdala, motor cortex and hypothalamus of the binge sucrose rats, much as seen in other studies investigating the effects of glycaemic fluctuations in diabetes^39^ and high-fat diet regimen^40^. Interestingly, the authors of those studies interpreted the microglial proliferation and activation as a homeostatic mechanism to improve glucose tolerance and insulin sensitivity and to allow regulation of glucose homeostasis. Indeed, microglia have a bivalent functional phenotype, such that microgliosis can occur without neurodegeneration^41^. Furthermore, the sucrose-diet had no corresponding comparable effect on astrocyte staining area in any brain region, except for a slight reduction in the hypothalamus (where astrocytes may mediate glucose-stimulated activation of pro-opio-melanocortin (POMC) neurons^42^). Thus, the sucrose diet-induced increase in [^18^F]DPA714 BP_ND_ likely reflects microglial activation and proliferation rather than changes in astroglial TSPO expression. Compilation of our PET and immunohistochemical findings supports a rough calculation of their relationship; an increase of 0.1 units of [^18^F]DPA714 BP_ND_ above baseline corresponds to a numerical increase of about 2000 microglia per mm^3^.

Remarkably, we also detected significantly elevated peripheral WBC counts in the binge sucrose rats, which may be indicative of a systemic inflammatory process. Western diets high in fat and sugar, especially fructose, can cause non-alcoholic fatty liver disease (NAFLD) and exacerbate other chronic liver disease. NAFLD, considered the hepatic manifestation of metabolic syndrome and type II diabetes, is associated with low-grade peripheral inflammation^43–45^, but NAFLD may also occur with a sugar diet in the absence of weight gain^46^. However, our analysis of the livers of the sucrose diet rats revealed neither abnormal liver histology nor any deposition of fat droplets. Furthermore, IBA-1 staining of the liver indicated no unusual macrophage aggregation or infiltration. This may not, however, preclude the presence of more subtle hepatocyte injury and liver inflammation; other possible mechanisms possibly contributing to the apparent systemic inflammation provoked by high sucrose diet include high fructose induced-elevated blood pressure, elevated serum triglycerides, or insulin and leptin resistance, which we did not assess in this study. Furthermore, the parallel increases in WBC counts and upregulated microglial markers may reflect the distinction between the metabolic effects of dietary sucrose and obesity per se, which may preferentially activate inflammatory processes in peripheral tissues^10,11^. Moreover, high-sucrose diets are known to lead to gastrointestinal dysbiosis, which can manifest as central and peripheral inflammation^47,48^ or can indirectly inhibit glutamine synthetase activity in peripheral and central immune cells^49,50^ to contribute to widespread inflammation. Although these and other putative mechanisms for sucrose-induced inflammation were not addressed in this study, the present findings of increased WBC counts and widespread microgliosis in brain of rats offered a binge sucrose diet has implications for the effects of the Western diet on brain health. Considering that LPS endotoxemia provoked an increased cerebral uptake of the beta-amyloid PET tracer [^18^F]flutemetamol in rats^51^ and given that LPS can interfere with hippocampal function^52^, one might speculate that the minor neuroinflammation evoked by the present binge sucrose diet might in the long term facilitate neurodegenerative processes.

## Supplementary Information

Supplementary Legends.

Supplementary Figure S1.
