# Supplementary material for: A binge high sucrose diet provokes systemic and cerebral inflammation in rats without inducing obesity
Source: Sci Rep. 2021 May 27;11:11252. doi: 10.1038/s41598-021-90817-z (PMC8160215; doi:10.1038/s41598-021-90817-z)
Supplement: Supplementary file 1 — Supplementary Legends. [file 41598_2021_90817_MOESM1_ESM.docx]

**Figure S1:** **Effect of 12-week sucrose intake on the liver.**

Representative images of H&E staining in the (A) liver of normal diet and sucrose diet rats, scale bar = 100 µm. (B) Image in A shown in high resolution, scale bar = 100 µm (C) Representative images of IBA-1 (macrophages) staining in the liver of normal diet and sucrose diet rats (green), scale bar = 200 µm.
