# Supplementary figures and images for: A binge high sucrose diet provokes systemic and cerebral inflammation in rats without inducing obesity
Source: Sci Rep. 2021 May 27;11:11252. doi: 10.1038/s41598-021-90817-z (PMC8160215; doi:10.1038/s41598-021-90817-z)

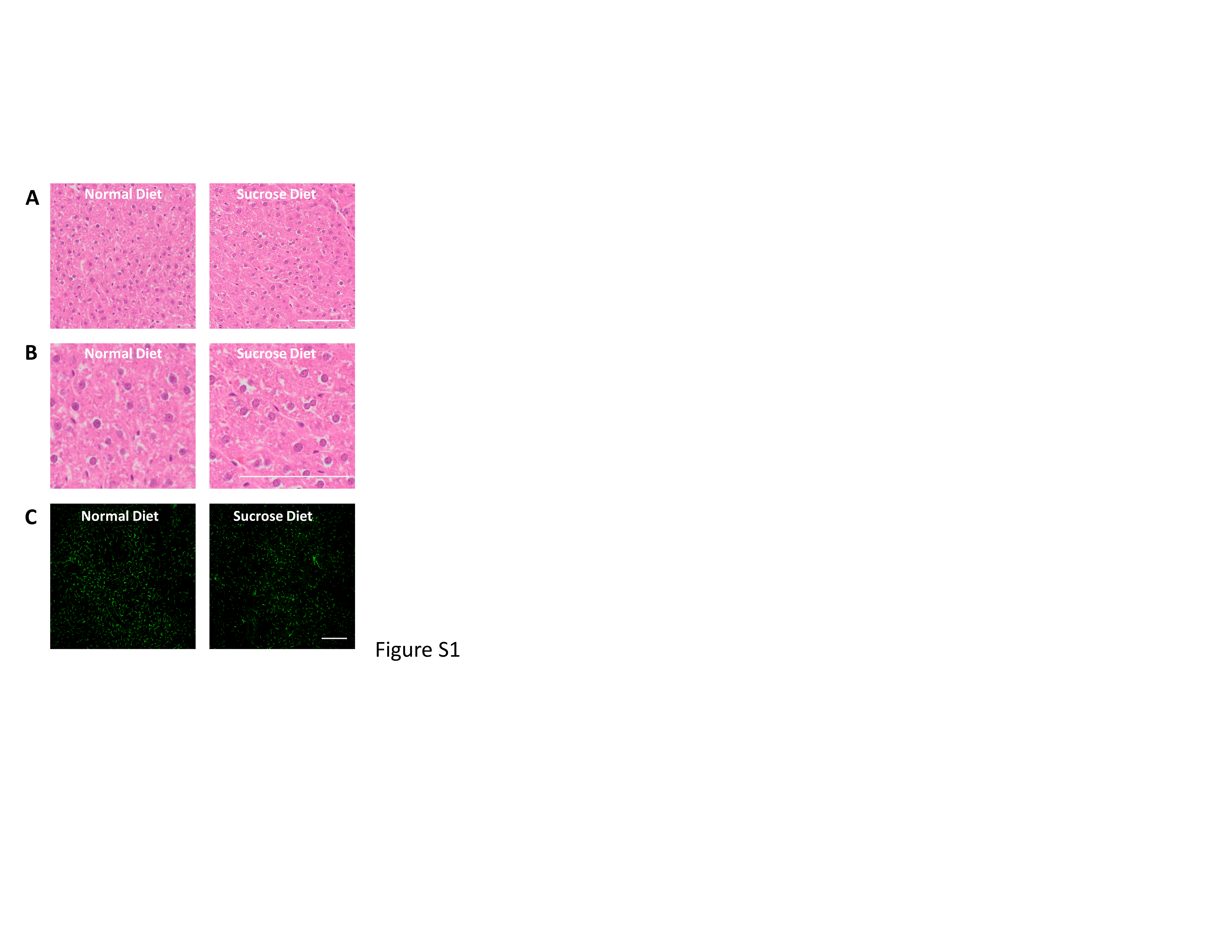

Supplement: Supplementary file 2 — Supplementary Figure S1. [file 41598_2021_90817_MOESM2_ESM.tif]
